# Blue plaque review series: Thomas Graham Brown: Before his time

**DOI:** 10.1113/EP093413

**Published:** 2026-04-15

**Authors:** Ronald L. Calabrese, Eve Marder

**Affiliations:** ^1^ Department of Biology for Calabrese Emory University Atlanta Georgia USA; ^2^ Department of Biology for Marder Brandeis University Waltham Massachusetts USA

**Keywords:** central pattern generator, half‐centre, oscillatory neuronal network, reciprocal inhibition

## Abstract

Thomas Graham Brown made a seminal discovery, published in 1911 while he was a Carnegie Fellow in the University of Liverpool laboratory of Nobel Prize winner Charles S. Sherrington. Working in cats, he showed that rhythmic ‘voluntary’ behaviour, such as stepping and, by inference, walking, does not result from a chain of reflex events, but that there are rhythmic circuits (oscillators) in the spinal cord that can produce a programme of alternating antagonistic muscular contractions and, by inference, motor neuron activities that underlie rhythmic stepping. This was the first definitive demonstration of what today we call a central pattern generator for an ‘important’ non‐automatic rhythmic behaviour, and it should have rocked the foundations of the Sherringtonian view of the nervous system, but it didn't. Like the biblical ‘*Vox clamantis in deserto*’ (Isaiah 40:3, Biblia Scara Vulgata), Brown was largely ignored by his contemporaries, including Sherrington, only to be rediscovered starting in the 1960s. The legacy of Thomas Graham Brown is not only his scientific contribution that ultimately impelled the field of motor pattern generation forwards but also an object lesson in how powerful voices can drown out the dissenters. It remains as a lasting imprint on our science.

## Abstract (highlights)

- **What is the topic of this review?**
  We review the seminal findings of the pioneer movement neuroscientist Thomas Graham Brown.
- **What advances does it highlight?**
  We recount how these findings were largely ignored for >50 years, then to be recognized as the first demonstration of a centrally programmed voluntary motor pattern. We discuss his findings in the context of our current understanding of half‐centre oscillators as the rhythm generators of central pattern generators.

## 1 INTRODUCTION

When discussing whether coordinated behaviours, such as walking and searching in crayfish, arose from sensory input, the famous 19th century scientist and philosopher T. H. Huxley (1880) wrote:

To carry the analogy of the musical instrument further, striking a single key gives rise, not to a single note, but to a more or less elaborate tune; as if the hammer struck not a single string, but pressed down the stop of a musical box.

It is in the ganglia that we must look for the analogue of the musical box. A single impulse conveyed by a sensory nerve to a ganglion, may give rise to a single muscular contraction, but more commonly it originates a series of such, combined to a definite end.

This view collided with those of the prevailing neurophysiologists of the next generation, most notably Sir Charles Scott Sherrington, whose landmark work, *The Integrative Action of the Nervous System* (1906), dominated thinking on motor control. To Sherrington, movement ultimately originated from reflex action: striking a single key gave rise to a single note, and the sounding of that note (movement) gave rise to new sensory input and reflex action. Sequential actions, even purposeful ones, could be viewed as a ‘chain of reflexes’. In 1932, Sherrington was awarded the Nobel Prize in Physiology or Medicine. The citation read, ‘In the 1890s Charles Sherrington showed how muscular contractions are followed by relaxation and how different reflexes are part of a complicated interplay in which the spinal cord and brain process nerve impulses and turn them into new impulses to muscles and organs’ (https://www.nobelprize.org/prizes/medicine/1932/sherrington/facts/).

Given this context, the work of Thomas Graham Brown (1882–1965) was initially neglected as bucking the prevailing wisdom, but ultimately recognized half a century later, such that his seminal experiment is today viewed as foundational in our understanding of motor control. It seems likely that Sherrington played at least a passive role in this neglect by not championing the work of his mentee and colleague, and thus he might be viewed as retarding progress in the field. Sherrington's contributions to the early science of the nervous system were, nevertheless, outstanding: precisely defining spinal reflexes in mammals and giving rise to ideas so seminal as inhibition and its balance with excitation. Moreover, his trainees include luminaries of early to mid‐20th century neuroscience: Wilder Penfield and Nobelists Sir John Eccles, Ragnar Granit and Howard Florey; indeed, even Thomas Graham Brown. Thomas Graham Brown thought highly of the integrity and kindness of Sherrington as a mentor, as evinced by his written tribute to Sherrington on his 90th birthday (Brown, 1947), although at this time Brown's work had little impact.

Much has been already written about Thomas Graham Brown's professional and personal history. These works include a biographical memoir by Lord Edgar D. Adrian (1966), a co‐1932 Nobel Laureate and neuroscience colleague of Sir Charles S. Sherrington, and pieces by Douglas G. Stuart and colleagues (Jones et al., 2011; Stuart & Hultborn, 2008; Wetzel & Stuart, 1976). These authors have endeavoured to explain Graham Brown’s fall‐off in research publication with his assumption of a position as Professor of Physiology in Cardiff (1920–1947), his turn to mountaineering as his principal interest, and the relative neglect of his remarkable opus of 1909–1920 owing to the lack of full‐throated support by Sherrington. Here, we hope to touch on these tragedies only peripherally and to focus on how Graham Brown's ideas were seminal to our own published work and current concepts of the control of locomotion and other rhythmic motor patterns. Suffice it to say here that Graham Brown was a Carnegie fellow in the laboratory of Sherrington at the University of Liverpool from 1910 to 1913, and that his most impactful experimental work originated at that time.

## 2 INTRINSIC FACTORS IN THE ACT OF PROGRESSION

**FIGURE 1:**
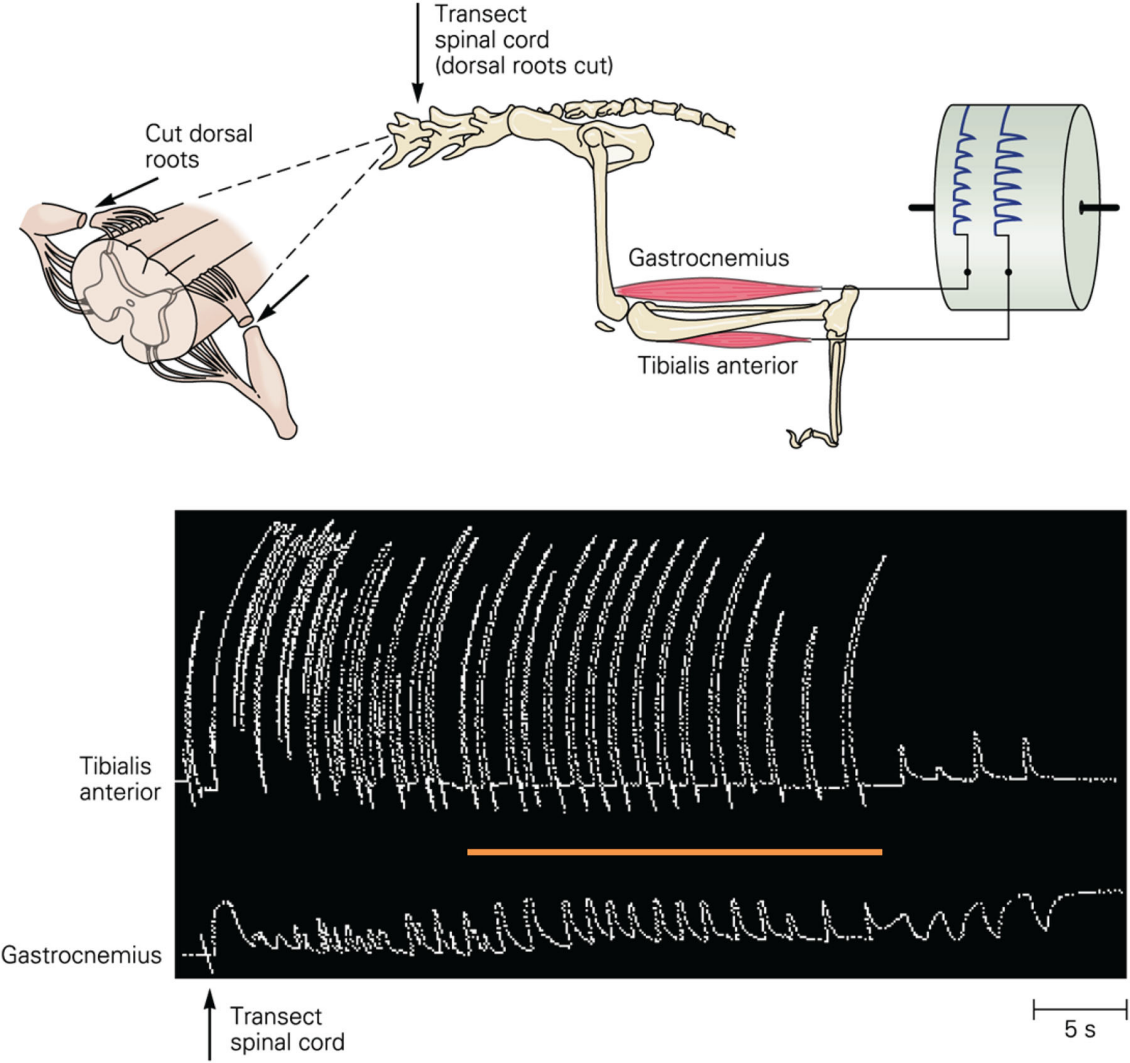
Rhythmic progression in a deafferented spinal cat induced by transection of the spinal cord immediately anterior (T12) to the lumbar enlargement. One hindlimb was prepared by cutting all motor nerves and/or removing muscles except tibialis anterior and gastrocnemius (ankle flexor and extensor, respectively). These still innervated muscles had their tendons cut and were attached by thread and levers to a kymograph drum to record their movements. All dorsal roots to L6 on both sides were cut. When the spinal cord was transected, initial predominant flexion gave way to an episode of progression (rhythmic flexor–extensor alternation, highly similar to progression in afferented preparations; orange bar), then to predominant extension. Published with permission from *Principles of Neural Science*, 4th Edition. Eric R. Kandel, James H. Schwartz, Thomas M. Jessell, 2000, McGraw‐Hill, New York, United States of America 0‐8385‐7701‐6 and Graham Brown, T., 1911c. The intrinsic factors in the act of progression in mammals. *Proc. R. Soc. B 84*, 308–319.

By the time Graham Brown joined Sherrington's laboratory, he had published (Brown, 1911a, 1911b, 1911c, 1912) on the scratch reflex in guinea pigs and rabbits and had shown that the scratch reflex can be evoked in deafferented preparations. He also noted the similarity of this reflex to progression, the then prevailing term for stepping in mammals. This led him to wonder, in the introduction of the seminal publication Brown (1911c) whether ‘… the act of progression may, too, be essentially a central and not a peripheral phenomenon.^1^ Figure 1 shows Graham Brown's definitive experiment. He immediately saw the implications of this experiment; he had ‘pressed down the stop of a musical box’. His own words describe the implications.

Summary By means of a stimulus (namely, section of the spinal cord) central in application, although remote from the local centre, the act of progression may be induced in muscles de‐afferented by the cutting of their appropriate posterior spinal roots. It occurs thus after all the muscles of both hindlimbs have been de‐afferented, and all but the recording pair have been put out of action by motor paralysis.

The act of progression as exhibited by these muscles and thus induced scarcely differs, if indeed it differs at all, from the act similarly induced when the afferent arcs of the recording muscles have not been broken.

In either case the reaction, as evidenced in movement at the ankle‐joint, shews three periods. In the first the record is characterized by a state chiefly of maintained flexion. In the last there is a state characterized by maintained extension. Intermediate between these there is a period of ‘balance’, in which the movements of progression are most perfect.

The rhythmic sequence of the act of progression is consequently determined by phasic changes innate in the local centres, and these phases are not essentially caused by peripheral stimuli.

The proprioceptive stimuli which are generated by the contraction of muscles taking part in the act (when the appropriate posterior spinal roots are intact) play a regulating and not an intrinsic part in the act. Their chief importance may be in the grading of the individual component movements to the temporary exigencies of the environment.

This was the first definitive demonstration of what today we call a central pattern generator (CPG) for an ‘important’ non‐automatic rhythmic behaviour, and it should have rocked the foundations of the Sherringtonian view of the nervous system, but it didn't. In fact, in a contemporaneous report, Sherrington (1913) twists himself into logical knots to explain away these results.^2^ Today, it is clear that rhythmic ‘voluntary’ behaviour, such as stepping and, by inference, walking, does not result from a chain of reflex events; there are rhythmic circuits (oscillators) in the spinal cord that can produce a programme of alternating antagonistic muscular contractions and, by inference, motor neuron activities, that underlie rhythmic stepping. Graham Brown recognized that such a programme might suffice for a cat walking over an even surface but would absolutely require reflex involvement to tune it to predatory stalking over rough terrain (Brown, 1911c).

Graham Brown also envisioned reciprocally inhibitory centres (that he termed half‐centres) in the spinal cord that provided for the flexor/extensor and left/right alternations important for stepping and that subserved reflexes. He also realized that such an arrangement is not necessarily oscillatory.

Brown's further work in Sherrington's laboratory explored rhythmic stepping in deafferented spinal cats and guinea pigs under anaesthetic narcosis (narcosis progression) (Brown, 1913a) and observed that progression continued in one hindlimb after ablation of the contralateral half of the lumbar spinal cord. This observation suggested to Brown that there were reciprocally inhibitory centres for each limb. He also noted the similarity between the progression he observed in deafferented preparations and the automatic rhythm of respiration, and he suggested that they might share the same underlying mechanism. Brown continued his work at University of Manchester, reporting further on narcosis progression in cats (Brown, 1914) and progression in cat fetuses (Brown, 1915), which reinforced his notions about half‐centre organization for progression and suggested that this was an innate rather than learned organization.

In 1915, Brown's research went into abeyance because he served in World War I until the end of 1919. His subsequent published research was not on progression, and much of his behavioural research on cat walking remained unpublished (Stuart & Hultborn, 2008). Remarkably, during the war he published a definitive two‐part review of his research and ideas about the reflexes and rhythmic activities in mammals (Brown, 1913b, 1916). The turmoil of war and the fact that this review was published in German in a German journal undoubtedly contributed to its obscurity.

**FIGURE 2:**
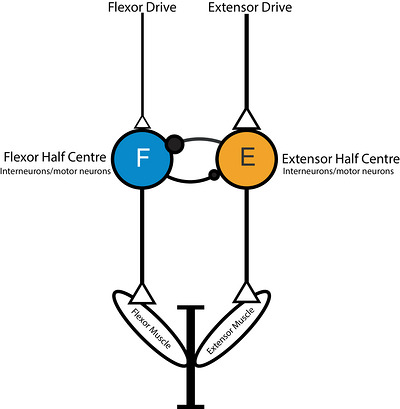
Graham Brown's half‐centre model for the control of stepping. This diagram is redrawn from figure 84 of Brown (1916). It shows his conception of reciprocal inhibition between a flexor (F) half‐centre and an extensor (E) half‐centre (both composed of a motor neuron and/or interneurons), in a phase of the step in which extension is occurring. The size of each synaptic ending (open triangles, excitatory; filled circles, inhibitory) indicates the strength of the connection and the extent of fatigue. Fatigue was not directly tied to synaptic depression but more likely to be accounted for by what today we would term spike‐frequency adaptation. Symmetry between half‐centres is not necessary for oscillator function.

Figure 2 represents the apotheosis of Graham Brown's half‐centre model for rhythmic activity. The model is agnostic as to whether the flexor and extensor half‐centres are composed of motor neurons, then very poorly studied/known interneurons, or a combination of the two. It is also agnostic about how the reciprocal inhibition is achieved by the half‐centres. To account for the asymmetry in the step cycle (swing–flexor phase vs. stance–extensor phase) in this word model, excitation to the extensor half‐centre is stronger, as is inhibition of the flexor half‐centre.

At this stage, it is informative to explore how prevailing thinking about the central generation versus reflex control of rhythmic behaviour evolved in the wake of Graham Brown's remarkable discovery. Contemporary neglect is certainly correct, but the ‘why’ is less clear. Clearly, Sherrington (1913) and Adrian (1931; Adrian & Buytendijk, 1931) knew that the respiratory rhythm was centrally generated, and they were well aware of Graham Brown's discoveries, but they seemed to ignore Brown's findings. As Graham Brown's research career wound down, he faded from the scene. Graham Brown was at this point a devoted mountaineer and was remembered after his death by Adrian (1966) as having lost his way in research; little notice was given to his half‐centre concept. This wind‐down provides in itself little reason for his work falling into neglect, and Stuart and colleagues provide some insights into reasons behind this fall (Jones et al., 2011; Stuart & Hultborn, 2008).

The chain of reflex ideas for rhythmic behaviours that Sherrington preferred prevailed through the 1950s and were championed by Sir James Gray at Cambridge. Gray, Lissmann and Pumphrey argued that the swimming rhythm of the leech was reflexly programmed despite the evidence largely favouring central origin (Gray et al., 1938). Earlier, the famous German behavioural biologist Erich W. von Holst (1937) argued for the central origin of rhythmic fin movements in fish. Respiration in both vertebrates (as noted by Graham Brown; see above) and invertebrates was widely accepted as being generated by central oscillatory circuits (Adrian, 1931; Hoyle, 1959; Miller, 1960a). Indeed, Adrian himself had recorded the central respiratory rhythm of goldfish (Adrian, 1931). Gray went on to study terrestrial locomotion in toads, mapping out reflexes that could account for the locomotory behaviour, and his work largely dominated thinking. His colleague at Cambridge, Lissmann, who studied dogfish swimming, provided evidence for some degree of central patterning while insisting that the rhythm was not of central origin (Lissmann, 1946). Gray's own work with Lissmann on frog reflexes in locomotion (Gray & Lissmann, 1946) and leech swimming (Gray et al., 1938) also pointed in this direction. In 1950, an influential Symposium of the Society of Experimental Biologists was held that highlighted Gray's (1950) views but included dissenting voices. The noted developmental biologist Paul Alfred Weiss, then at the University of Chicago, argued using supernumerary limb grafts that frog terrestrial locomotion is controlled by a central rhythm associated with central connections (Weiss, 1950). This background set the stage for the ‘rediscovery’ of Graham Brown.

Conceptual shifts in scientific thinking, like revolutions, begin with a few dissenting voices and build to a tipping point. That tipping point came in 1961 with the publication of Donald Wilson's experiments on locust flight, in which he showed that after removal of all afferent input from the wings, the animal could still produce coordinated rhythmic wing beats with only a wind stimulus to head mechanoreceptors, albeit with a reduced frequency from normal flight (Wilson, 1961). The discussion section of this classic paper is worth reading, because it succinctly summarizes the research on motor rhythm production and comes squarely to the same conclusion as Graham Brown; i.e., the basic rhythm and its coordination are centrally generated, and sensory input plays a regulatory role adjusting movement to the exigencies of the environment.

There are two ironies in Wilson's paper: first, his original aim had been to show that wing proprioceptors played a critical role in generating the flight rhythm (personal communication to R. L. Calabrese); and second, that although he cites evidence for central generation of motor rhythms in other systems, he does not cite Brown (Wilson, 1961). This was followed by the study by Wilson & Wyman (1965), in which the wind stimulus needed for generation of the flight rhythm in a deafferented preparation was replaced by random electrical stimulation of the nerve cord in decapitated animals. Wilson's laboratory also showed that input from the wing proprioceptors is necessary to attain normal wingbeat frequency in animals activated by a wind stimulus to the head, but this input need not timed to the wingbeat cycle (Wilson & Gettrup, 1963). The paper by Wilson & Wyman (1965) marks the full shift in Wilson's thinking. The term ‘central pattern generator’ probably arises from this work as it embraces the central (in most cases, premotor) circuitry that produces a rhythmic motor pattern.

In 1960, Hughes and Wiersma published their studies on the swimmeret system of crayfish, providing clear evidence of central generation of the rhythm; but still Hughes, a past and future colleague of Gray at Cambridge, would not go against his mentor. In the discussion (Hughes & Wiersma, 1960) he writes:

The observations described above are of interest in relation to the much‐discussed problem of whether rhythmic movements are primarily due to occurrences in the C.N.S. or are triggered by peripheral sensory inflow. Rhythmic electrical activity in the deafferented C.N.S. has been considered as favouring the first viewpoint (Adrian, 1931; von Holst, 1937). On the other hand, evidence has been obtained that in order to make coordinated limb movements possible, at least one limb must have both afferent and efferent innervation intact (Gray, 1950) and a similar finding was made with respect to the leech, in which rhythmic electrical discharges were present in the cord as long as a limited region of the body was connected to it, whereas after isolation of the cord no comparable rhythmicity obtained (Gray et al., 1938). Our results on the isolated abdomen are in fairly good agreement with this latter view, for the rhythmic discharges which can be led off from a preparation with only a single first root intact are essentially similar to those of the intact cord; but this is not true when this last remaining root is severed. It is thus evident that proprioceptive impulses are of importance for normal coordination of the swimmerets under these conditions.

Wilson perhaps saw this for what it was and listed the Hughes & Wiersma (1960) paper as supporting in general, if not wholly, central motor rhythm generation. This ‘counter example’ did not stand long. Ikeda & Wiersma (1964) showed that properly maintained isolated nerve cords could produce the complete intersegmentally coordinated swimmeret rhythm spontaneously. Unfortunately for Wiersma, Wilson (1961) caused the conceptual shift, and Wiersma's contribution was not as impactful. There are, however, dissenting voices on the ultimate impact of Hughes and Wiersma's experiments (Mulloney & Smarandache, 2010).^3^

Important work was also occurring in vertebrates, from amphibians to mammals, that further established that locomotor patterns were centrally generated (Grillner & Wallen, 1985). Of particular note, starting in the 1970s, Grillner and colleagues, building on earlier work by Roberts (1969) [ironically, Roberts, like Lissmann (1946) before him in dog fish and Hughes & Wiersma (1960) in crayfish, concluded that afferent input was required for motor pattern production despite his data], established that the swimming in the spinal dogfish was centrally generated (Grillner et al., 1976). Then, building on the pioneering work of Rovainen (1967a, 1967b), they began an in‐depth analysis of the central circuitry underlying swimming in the lamprey, resulting in this primitive vertebrate being one of the best understood CPG in vertebrates to date (Grillner, 1985; Grillner et al., 1981, 1991, 1983).

Seminal work by Donald Maynard, Allen Selverston and colleagues (Dando & Selverston, 1972; Hartline & Maynard, 1975; Maynard, 1966, 1972; Maynard & Dando, 1974; Miller & Selverston, 1982a, 1982b; Mulloney, 1977; Mulloney & Selverston, 1974a, 1974b; Selverston & Miller, 1980; Selverston & Mulloney, 1974; Selverston et al., 1976) brought the stomatogastric nervous system of Crustacea to the fore as a premier preparation for studying the central generation of motor rhythms. The stomatogastric nervous system comprises two relatively independent pattern generators controlling the pylorus and the stomach teeth (gastric mill). The pyloric network is spontaneously active continuously and driven by a bursting interneuron (AB), hence early work focused on this system did not seem a good model for episodic half‐centre‐based rhythms, such as the gastric mill rhythm and locomotion. The connectivity of the episodic gastric mill was established early (Mulloney & Selverston, 1974a, 1974b; Selverston & Mulloney, 1974), but an understanding of how rhythmicity arose from this connectivity was complicated by the fact that there are different forms of the gastric mill rhythm (Heinzel, 1988a, 1988b). Even in the earliest studies on the gastric mill, it was thought that the reciprocal inhibitory connections between the interneuron 1 (Int1) and lateral gastric neuron (LG) neurons were key.

By 1983, the conceptual change arguing that rhythmic motor patters were centrally generated was complete, as evinced by a Symposium of the Society of Experimental Biologists on ‘Neural Origin of Rhythmic Movements’ (Roberts & Roberts, 1983).^4^ This volume covers a wide diversity of systems producing centrally programmed motor programmes in arthropods, molluscs, leech, amphibians, lamprey and mammals. Graham Brown's essential and early contribution was recognized in several papers. Moreover, an influential review by Delcomyn (1980) puts Graham Brown's work into the context of the plethora of centrally programmed behaviours.

Stuart & Hultborn (2008) argue convincingly that the work of Anders Lundberg and his collaborators, especially Elzbieta Jankowska et al. (1965), brought Graham Brown's findings and the half‐centre concept into the mainstream of vertebrate spinal cord research. Lundberg's research had focused on the hunt for the spinal half‐centres that produce stepping, a hunt that, despite years of progress and the advent of genetically identifiable cell types, remains elusive (Arber et al., 2000; Jessell et al., 2011). Thus, while the invertebrate research community was complicit in ignoring Graham Brown's work, they were in the hunt for half‐centre CPGs with Lundberg. See Lundberg (1981) for his views on role of half‐centre oscillators in rhythmic stepping.

Above, we have neglected the discussion that Graham Brown provides about how a set of reciprocally inhibitory half‐centres can become oscillatory, but it is this discussion that has conditioned our own research. As we started our research careers in the late 1960s and early 1970s, we needed no convincing that CPGs existed; we wanted to know how their rhythms and phase relationships were generated in terms of cellular and synaptic mechanisms [Wilson's work had entered the realm of undergraduate education with a *Scientific American* article (Wilson, 1968)]. Workers in small rhythmic circuits from various species focused on identifying the CPG network neurons and their synapses and were, perhaps, overly optimistic that this work would find a few general mechanisms that held across preparations (Selverston, 1980). The realization that there were CPGs not driven by endogenous bursters, especially those for episodic behaviours such as locomotion versus continuous ones such as respiration, and the finding of reciprocal inhibition as a widely used building block in these networks focused interest on this motif as a potential oscillatory core of CPGs (Getting, 1989). Theory initially led the way, as physiological analyses of the intrinsic membrane properties of the component neuron and their synaptic connectivity intensified. How could reciprocally inhibitory neurons (half‐centres) generate rhythmic activity? Why didn't one half‐centre dominate the other stably?

Graham Brown wrestled with ideas about the origin of oscillation and wrote critically in all his papers on progression about potential mechanisms. He knew that the key to understanding the genesis of half‐centre‐driven alternations between functional antagonists depended on accounting for the mechanisms underlying the transitions between the off and on states of activity in the constituent neurons. He argued (Brown 1914, 1916) that excitation, provided extrinsically (e.g., by balanced sensory input or severance of the spinal cord; Figure 1), and inhibition in the system must be in a functional balance. Furthermore, he realized that oscillation demanded either that the inhibition evoked by the firing half‐centre must fatigue, releasing the other half‐centre to fire, or that excitation, perhaps brought on by rebound, might allow the inhibited half‐centre to begin to fire, suppressing the other half‐centre, or a combination of the two. Rebound as a concept is tricky in Graham Brown's work, because it is tied to reflex rebound rather than a more current understanding of cellular rebound after synaptic inhibition. Indeed, Graham Brown had little cellular understanding of inhibition: ‘…in this scheme no explanation of the inhibitory phenomenon is given (Brown & Sherrington, 1912)’. It is notable that he argued against the notion that each half‐centre was endogenously rhythmic.

Using digital simulations and analog neuron models (neuromimes), Harmon (1959) and Reiss (1962) explored how reciprocally inhibitory neuron pairs could produce oscillatory output and essentially came to the same conclusions as Graham Brown; i.e., tonic excitation and adaptation of firing (fatigue) could result in rhythmic alternation in firing. It is interesting that Reiss (1962) did not seem to be aware of Wilson's or Graham Brown's findings and seemed mainly motivated by building autonomous machines. Harmon (1964) followed up with neuromime studies that showed that self‐inhibition could support oscillation in reciprocally inhibitory neurons. Harmon does attribute his motivation for studying the role of reciprocal inhibition in oscillation to Wilson's (1961) speculations on the locust flight systems, but, like Reiss, he was unaware of Graham Brown and attributed the interest in the motif for controlling antagonistic muscular systems to McDougall (1903) and Sherrington (1906).^5^ Szekeley (1965) broke entirely from the half‐centre concept and proposed that unidirectional rings of odd numbers of inhibitory premotor neurons could account for locomotion in salamanders. Wilson & Waldron (1968) followed up with their own neuromime studies, trying to capture nuances of the wingbeat motor pattern of locust flight and exploring layered reciprocally inhibitory neurons, again with no reference to Graham Brown. Perkel & Mulloney (1974) explored the role of cellular rebound from inhibition in producing oscillation in reciprocally inhibitory neurons, again with no reference to Graham Brown. Thus, theoretical exploration of half‐centre oscillators was proceeding apace in the apparent absence of awareness of Graham Brown's ideas.

As physiological analyses of invertebrate CPGs continued in the 1980s and 1990s, Graham Brown's ideas crept into the awareness of researchers, facilitated by Declomyn's review (1980). Stent et al. (1978) made progress on identifying the neurons of the leech swimming CPG and produced model circuits (neuromimes) based on the known synaptic connectivity of interneurons and motor neurons and Szeleley's ideas about ring structures (Szekely, 1965). Yet even here, reciprocal inhibition emerges in the connectome. The flood gates had opened, and system after system that was explored yielded to cellular analyses of a CPG, as evinced by the Symposium of the Society of Experimental Biologists on the ‘Neural Origin of Rhythmic Movements’ (Roberts & Roberts, 1983), with reciprocal inhibition a common theme.

A few admittedly biased highlights seem worth mentioning explicitly as they relate to Graham Brown's half‐centre hypothesis. Getting and co‐workers cracked the swim CPG of the mollusc *Tritonia* (Getting, 1983), in which reciprocal inhibition plays a role, and Getting wrote a highly influential review (Getting, 1989) highlighting the role of reciprocal inhibition in pattern generation. The connectivity of the crustacean stomatogastric nervous system is dominated by reciprocal inhibitory synapses and electrical coupling (Marder & Bucher, 2007). A large number of descending modulatory neurons provide excitation to both the gastric mill and pyloric networks (Bartos & Nusbaum, 1997; Bartos et al., 1999; Beenhakker et al., 2004, 2005; Blitz & Nusbaum, 1999; Blitz et al., 1999; Nusbaum & Beenhakker, 2002; Nusbaum et al., 2017), and much of the circuitry involves activation and coordination of reciprocally inhibitory neurons. Moreover, recent modelling work of interactions between fast and slow half‐centres provides insight into how neurons might switch between fast and slow rhythms (Gutierrez & Marder, 2013, 2014; Gutierrez et al., 2013). All these studies demonstrate that half‐centre interactions can be used as components in more complex circuits, capable of rich dynamics.

It became clear that the CPG driving the intersegmental vascular rhythm (heartbeat) of leeches was powered by two symmetric half‐centre oscillators (premotor interneurons) coupled by coordinating interneurons (Calabrese & Peterson, 1983). Arbas & Calabrese (1987a, 1987b) presented evidence that the hyperpolarization‐activated inward current, *I*_h_, provided a mechanism by which one member of a half‐centre oscillator might escape the inhibition of the other. There was a renewed interest in half‐centre oscillators and the role of *I*_h_.

Wang & Rinzel (1992) led with an analysis of escape and its counterpart release in producing oscillation in half‐centre oscillators. Release occurs when the inhibitory influence of the firing neuron wanes as its activity wanes, such that it moves below its threshold for synaptic transmission (the fatigue of Graham Brown), and escape occurs when the inhibited neuron can begin to fire such that it moves above its threshold for synaptic transmission before the opposing neurons activity ends (as proposed by Arbas & Calabrese, 1987a, 1987b). Skinner et al. (1994) refined this theory by emphasizing that the escape or the release could be mediated by intrinsic or synaptic factors. Taylor et al. (2002) explored unique aspects of half‐centre oscillators based on inhibitory synaptic depression. For the leech heartbeat half‐centre oscillators, a series of modelling studies (Hill et al., 2001, 2002, 2003; Nadim et al., 1995; Olsen et al., 1995) established that this oscillator functions with both escape driven by *I*_h_ and release as firing wanes during a burst attributable to inactivation of an underlying slow Ca^2+^ current. These models were based fully on biophysically measured synapses and intrinsic currents and thus might be the most realistic half‐centre oscillator model even now.

**FIGURE 3:**
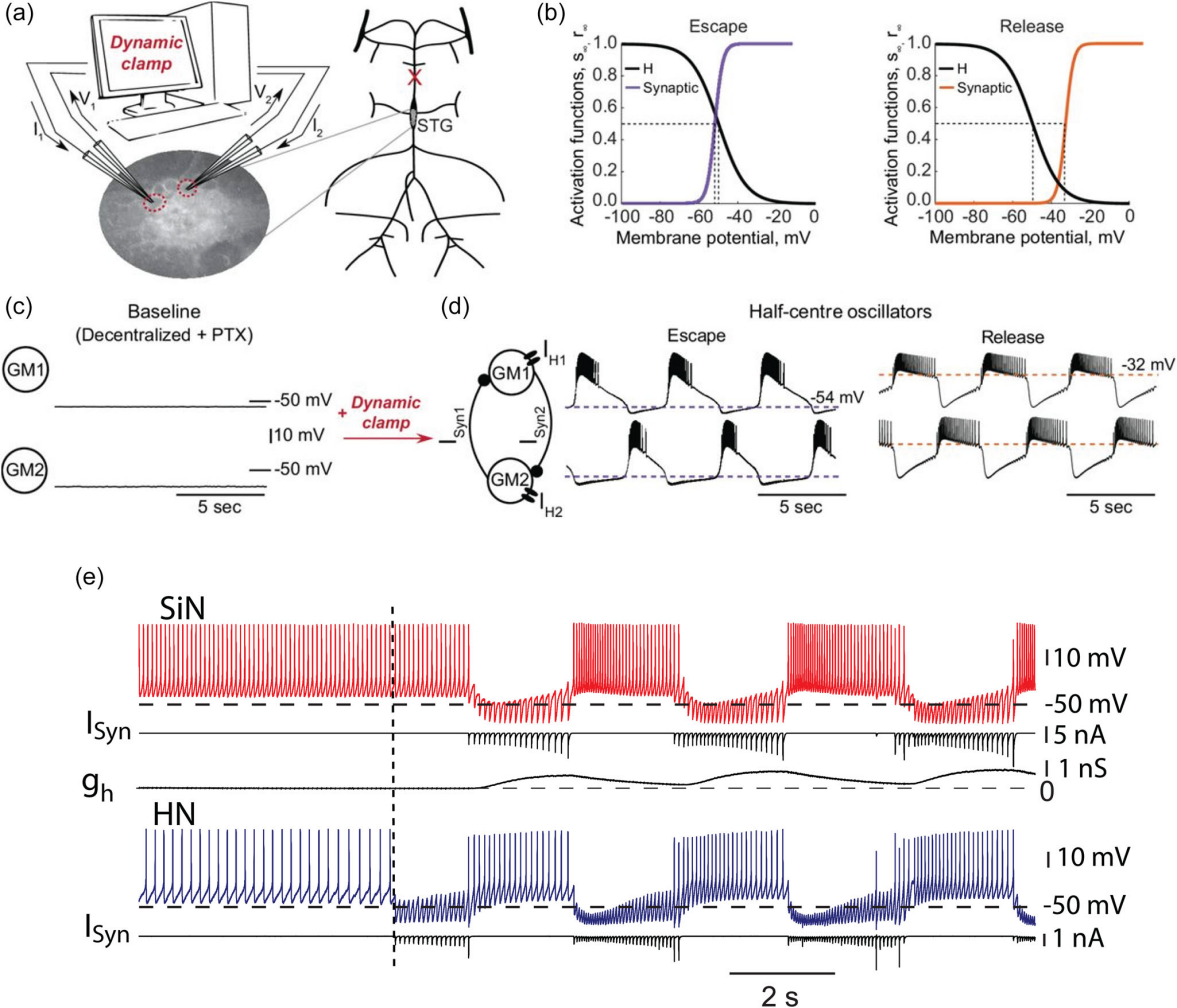
(a) Half‐centre oscillator circuits are built by connecting two gastric mill (GM) neurons from the stomatogastric ganglion (STG) of the crab *Cancer borealis* via artificial reciprocal inhibitory synapses (*I*_Syn_) and by adding an artificial hyperpolarization‐activated inward current (*I*_H_) in two‐electrode dynamic‐clamp mode using RealTime eXperimental Interface. The membrane potentials of the neurons (*V*_1_, *V*_2_) are digitized and passed to a computer to calculate the currents (*I*_1_, *I*_2_), which are then converted to analog signals and injected into the appropriate neurons. (b) Activation curves of the dynamic clamp‐generated hyperpolarization current and synaptic current. A shift in the synaptic activation curve switches the mechanism of oscillations between escape (left graph, purple curve) and release (right graph, orange curve). (c) At baseline, synaptically isolated GM neurons are silent, with a resting membrane potential between −65 and −55 mV. (d) When coupled via the dynamic clamp, the neurons generate an alternating bursting pattern of activity (half‐centre oscillator). Representative half‐centre oscillator traces with the escape mechanism are shown on the left and with the release mechanism on the right. Synaptic thresholds are indicated by the horizontal dashed lines. We can recognize an escape because the inhibited neuron begins its burst before the inhibiting neuron has descended below its synaptic threshold, and we can recognize a release because the inhibiting neuron descends below its synaptic threshold before the inhibited neuron begins its burst. In the circuit diagram, filled circles indicate inhibitory synapses. (e) Strong artificial inhibitory synapses cause an immediate transition from tonic firing to rhythmic bursting in a hybrid half‐centre oscillator made between a living HN neuron (part of a reciprocally inhibitory pair that forms a natural half‐centre oscillator driving the heartbeat central pattern generator in the leech), with its synaptic input blocked (bicuculline) and an analog very large scale integration silicon SiN neuron built to model a living HN neuron. Synaptic threshold was adjusted to −20 mV, assuring that only spikes mediated synaptic transmission. Membrane potential, synaptic current (*I*_Syn_) and h‐current conductance (*g*_h_) are shown for the SiN and *I*_Syn_ for the HN interneuron. The vertical dashed line indicates the time at which the dynamic clamp was activated to enable the reciprocally inhibitory synapses. Activating the synapses caused one of the neurons (HN) to be inhibited, while the other continued to fire tonically. Once a neuron was inhibited, its *I*_h_ was activated; meanwhile, the spike rate of the active neuron rapidly increased and then slowly decreased because of the activation and subsequent inactivation of a slowly inactivating Ca^2+^ current (*I*_CaS_). Once the spike rate in the active neuron was low enough, the inhibited neuron escaped from inhibition and began firing, inhibiting the other neuron. This process then repeated, resulting in antiphasic bursting. The living neuron relies on its endogenous *I*_h_ and *I*_CaS_ (no artificial current other than the synaptic current is added), and the SiN has a model *I*_h_ and *I*_CaS_ that are based on voltage‐clamp measurements in living HN interneurons. (a–d) Reproduced from Morozova et al. (2022). (e) From Sorensen et al. (2004), coloured and simplified for clarity.

Sharp et al. (1996) used the newly developed dynamic clamp technique (Sharp et al., 1993a, 1993b) to construct a hybrid half‐centre oscillator from rather generic stomatogastric ganglion GM motor neurons by adding artificial reciprocal inhibitory synapses and *I*_h_ to each cell. In the process, they verified the theoretical considerations of Skinner et al. (1994, 1993) and motivated others to use the dynamic clamp to verify their models of half‐centre oscillators experimentally (e.g., Olypher et al., 2006; Sorensen et al., 2004) and extend the analysis in the context of neuromodulation (e.g., Morozova et al., 2022; Figure 3a‐d). These dynamic‐clamp experiments show graphically how correct Graham Brown's thinking was about the forces that drive oscillation in reciprocally inhibitory half‐centres.

In essence, the field of motor pattern generation has moved on from the issues surrounding Graham Brown's seminal discovery and conceptual advance; these are now canon. Advances in molecular identification of neurons by transcription factor (Friese et al., 2009; Gatto et al., 2019; Lanuza et al., 2004; Paixao et al., 2019; Roome et al., 2020) and RNA profiling (Siletti et al., 2023; Sugino et al., 2019) now make cell identification possible in the vertebrate spinal cord and brain. Optogenetics (Buzsaki et al., 2015) and modern connectomics at the electron‐microscopic level (Bidel et al., 2023; Huang et al., 2021; Hulse et al., 2021; Scheffer et al., 2020; Schlegel et al., 2023) open up complex and simpler nervous systems to systematic analyses. Those studying motor control now perform cellular analyses with increasing precision (e.g., Singh et al., 2025) and make more elaborate circuit models with half‐centre cores (e.g., Rybak et al., 2024). Still, ‘the CPG’ for locomotion eludes us in vertebrates. In invertebrates, attention has shifted to how modulation and various perturbations influence and change motor networks (Ellingson et al., 2024; Li et al., 2018; Marder & Rue, 2021; Rue et al., 2022; Schapiro et al., 2024).

## 3 CONCLUSION

The legacy of Graham Brown is not only his scientific contribution that ultimately impelled the field of motor pattern generation forwards but also an object lesson in how powerful voices can drown out the dissenters. It remains as a lasting imprint on our science.

## AUTHOR CONTRIBUTIONS

Both authors conceived and wrote the work.

## CONFLICT OF INTEREST

None declared.

## Footnotes

1 In this very same introduction, Graham Brown (1911c) states that he and Sherrington ‘…demonstrated at a meeting of the Physiological Society a cat in which scratching may be elicited although both hind limbs have been completely de‐afferented’.

2 Speaking about the mechanisms of progression, Sherrington (1913) writes: ‘A fourth possibility is to regard, on the analogy of the respiratory centre, the rhythm of the step‐reflex as of essentially intrinsic origin; a natural and native rhythmic reply of the nerve‐cells to an inner stimulus such for instance as the blood. This is a view to which T. Graham Brown inclines, who, so far as he has at present elaborated it, bases it chiefly upon his observations of the rhythmic stepping obtainable by the mechanical stimulation of the spinal cord by section, and on the stepping, so to say, liberated by deep narcosis. It is a view that demands careful attention, but, as it seems to remove stepping from the category of reflexes except in so far as concerns regulation, the present argument which proceeds on the assumption that the forms of stepping here dealt with are reflex would be led too far afield by its consideration now’.

3 It is interesting to compare Donald Wilson and Graham Brown. Both were avid climbers, Graham Brown concentrating on the Alps and Wilson on the towering rocks of the American southwest. Both were iconoclastic in their lives and science, but while Graham Brown's light was hidden under a basket (not entirely of his own making), Wilson enjoyed scientific acclaim for similar discoveries and ideas. In both cases, their scientific careers were cut short, Graham Brown by a sort of self‐imposed exile at the University of Cardiff and Wilson by dying in a tragic rafting accident on the middle fork of the Salmon river in Idaho in 1970 aged 37. For more insights into the lives of these seminal figures, see Jones et al (2011) for Graham Brown and https://faculty.ucr.edu/~currie/donald‐wilson.htm for Donald Wilson.

4 It is informative in considering the scope of this conceptual change by 1983 to view the table of contents of Roberts & Roberts (1983): Ionic mechanisms of rhythmic firing / D. Noble Networks of local interneurons in an insect / M. Burrows and M.V.S. Siegler Cooperative mechanisms for the production of rhythmic movements / A.I. Selverston, J.P. Miller and M. Wadepuhl Neural control of swimming in Tritonia / P.A. Getting Neuronal mechanisms underlying rhythmic bursts in crustacean cardiac ganglia / K. Tazaki and I.M. Cooke Gastropod feeding: behavioural and neural analysis of a complex multicomponent system / P.R. Benjamin Neural control of heartbeat in the leech, Hirudo medicinalis / R.L. Calabrese and E. Peterson Hormonal control of the development and release of rhythmic ecdysis behaviours in insects / J.W. Truman and J.C. Weeks Neurons controlling the initiation, generation and modulation of leech swimming / W.B. Kristan and J.C. Weeks Initiation and control of swimming in amphibian embryos / A. Roberts, S.R. Soffe, J.D.W. Clarke and N. Dale The neural generation of locomotion in the lamprey: an incomplete account / S. Grillner, P. Wallén, A. McClellan, K. Sigvardt, T. Williams and J. Feldman Identified neurons in the lamprey spinal cord and their roles in fictive swimming / C.M. Rovainen Motor pattern formation in the dogfish spinal cord / B.L. Roberts and R.M. Williamson The control of rhythmic limb movements in Crustacea / W.J. Heitler The vertebrate scratch reflex / P.S.G. Stein Centrally generated pattern of motoneuron activity during locomotion in the cat / C. Perret Factors determining motoneuron rhythmicity during fictive locomotion / L.M. Jordan Supraspinal aspects of locomotion in the mesencephalic cat / S. Mori, K. Kawahara and T. Sakamoto On the origin and pattern control of breathing rhythmicity in mammals / C. Von Euler

5 From Harmon (1964): ‘The conjecture that reciprocally inhibiting pairs of neurons could be responsible for the control of antagonistic muscular systems was first made in 1903 by McDougall (1903). He proposed that the rhythmic alternate contractions and relaxations responsible for locomotion might arise from the action of pairs of single units with crossed inhibitory axon collaterals. Several years later Sherrington (1906) discussed similar notions’.
